# Supplementary material for: Biofilm formation, agr typing and antibiotic resistance pattern in methicillin-resistant Staphylococcus aureus isolated from hospital environments
Source: PLoS One. 2024 Aug 5;19(8):e0308282. doi: 10.1371/journal.pone.0308282 (PMC11299820; doi:10.1371/journal.pone.0308282)
Supplement: Attachment — Submitted filename: PLOS ONE revision.docx [file pone.0308282.s002.docx]

**Dear Editorial Board of the PLOS ONE journal,**

I have reviewed the manuscript entitled " Biofilm formation, agr typing and antibiotic resistance pattern in methicillin-resistant Staphylococcus aureus isolated from hospital environments " and would like to provide my feedback as follows:

Comment 1: Please delete the point at the end of the manuscript title.

Comment 2: In line 17, "MRSA's occurrence" should be replace with a better phrase, and the sentence should be rewritten.

Comment 3: In line 21, "PCR" should be written in full form for first time.

Comment 4: In line 21 and throughout the manuscript "mecA" should be written in italic form.

Comment 5: In line 25 and throughout the manuscript " icaA, icaB, and icaD" should be written in italic form.

Comment 6: In line 30, Please follow the order of the group II and III.

Comment 7: In line 22 and 23, please classified the isolates into biofilm forming and non-biofilm forming. Then the biofilm forming isolates classified into strong, moderate, and weak patterns. Please separate these two categories from each other's throughout the manuscript (following diagrams).

S. aureus isolates

Non-Biofilm forming

Biofilm forming

weak

moderate

strong

Comment 8: In line 39, please delete "Staphylococcus aureus" and only use "MRSA". The full name is already mentioned. Please check this point throughout the text.

Comment 9: In line 42, please replace "gram-positive" with "Gram-positive". Gram is the name of a scientist and must start with a capital letter.

Comment 10: In line 47, please add the comma after "communities".

Comment 11: In line 58, please delete "polysaccharide intercellular adhesin" and use "PIA".

Comment 12: In line 60, please replace "agr (accessory gene regulator)" with "accessory gene regulator (agr)". Please check all abbreviations and full names throughout the manuscript.

Comment 13: In line 63 and 64, please rewritten the sentence.

Comment 14: In line 71, please replace " *Staphylococci*" to "staphylococcal", in not italic form.

Comment 15: In line 86, please add the comma after " typing".

Comment 16: In line 103, please use "MSA" after "Mannitol Salt Agar".

Comment 17: In line 110, please specify genes required for approving S. aureus and MRSA isolates, separately.

Comment 18: In line 116, please replace " methicillin" with " oxacillin (1 μg) ".

Comment 19: In line 116, according to CLSI 2021, the approved method to detection of MRSA is: 1)MIC for oxacillin MIC, 2) MIC and disc diffusion for cefoxitin. Please justify this point for MRSA detection.

Comment 20: In line 124, please add "TSB" after "tryptic soy broth".

Comment 21: In line 132, after washing the formed biofilms, please explain what method was used to fixation them.

Comment 22: In line 135, please mention the manufacture company and other detail about of the device (ELISA reader).

Comment 23: In line 136, Please use the following formula to classify biofilms and change your data accordingly: OD < ODc (non-biofilm producer), ODc < OD < 2xODc (weak biofilm producer), 2xODc < OD < 4xODc (moderate biofilm producer), 4xODc < OD (strong biofilm producer).

Comment 24: In line 148: Please use the full name of "CLSI".

Comment 25: In section "Antibiogram Study of Isolated Biofilm-forming MRSA Strains", streptomycin is not recommended against S. aureus isolates according to CLSI guidelines. Please justify that why this antibiotic was chosen for antibiogram susceptibility test?

Comment 26: In section " Descriptive Analysis", please justify that why confidence intervals were considered 95%?

Comment 27: In section "Bivariate Analysis", please define other statistical tests used in the study, and more complete this section.

Comment 29: In line 172, please replace " Staphylococcus aureus" with "S. aureus".

Comment 30: In table 3 and 4, please define the "medicine ward". The urology and Surgery are not medicine ward??? Please also edit this point in figure 3, and throughout the manuscript.

Comment 31: In line 176 and 177, please define the final frequency of MRSA among S. aureus in this study. MRSA is confirmed by both disc diffusion and the presence of mecA gene.

Comment 32: In table 3 and 4, please justify that why P- value was calculated?

Comment 33: In line 187-190, please categorize the isolates firstly into biofilm forming and non-biofilm forming isolates. Then, the biofilm forming isolates should be classified into classes: strong, moderate, and weak. Please add a table about these categories (according to comment 7).

Comment 34: In tables 6 and 7, please add in a discription about statistical significance and confidence intervals (as described in table 3,4).

Comment 35: Please add a table about the prevalence icaA, B, C, D in different agr groups, which was one the main goals of this study. Please calculate the P-value about these data (as presented in table 6).

Comment 36: please justify that why authors did not determine the prevalence of MDR pattern among MRSA isolates. The investigation of relationship between MDR pattern and biofilm forming genes and agr system could more increase the scientific value of study.

Comment 37: In discussion, please deeply discus about the impact of agr system on biofilm-forming genes (icaA,B,C,D) and antibiotic resistance.

Comment 38: In my opinion, one of the main deficiencies in this article is the lack of attention to the expression levels of biofilm forming genes, which, if included, can have a significant impact on the scientific quality of the paper. Because presence or absence of these genes can influence S. aureus antibiotic resistance and biofilm formation, nonetheless, but the expression levels of these genes can vary due to environmental conditions and regulatory factors. Therefore, even if the genes are present, their expression levels can fluctuate, leading to differences in QS-mediated behaviors, such as biofilm formation and virulence factor production. To determine their expression levels in clinical strains of S. aureus, molecular techniques like quantitative gene expression analysis (qPCR) can be employed.

Comment 39: While the discussion highlights discrepancies in antibiotic resistance patterns and agr system, it does not thoroughly discuss the limitations of the study. It's essential to acknowledge potential limitations, such as sample size, selection bias, or methodological constraints, to provide a balanced interpretation of the results.

Comment 40: Consider adding a brief section in the discussion that outlines potential future research directions or practical implications of the study's findings. This can help readers understand the broader significance of the research.
